# Blood-Based Transcriptomic Biomarkers Are Predictive of Neurodegeneration Rather Than Alzheimer’s Disease

**DOI:** 10.3390/ijms241915011

**Published:** 2023-10-09

**Authors:** Artur Shvetcov, Shannon Thomson, Jessica Spathos, Ann-Na Cho, Heather M. Wilkins, Shea J. Andrews, Fabien Delerue, Timothy A. Couttas, Jasmeen Kaur Issar, Finula Isik, Simranpreet Kaur, Eleanor Drummond, Carol Dobson-Stone, Shantel L. Duffy, Natasha M. Rogers, Daniel Catchpoole, Wendy A. Gold, Russell H. Swerdlow, David A. Brown, Caitlin A. Finney

**Affiliations:** 1 Department of Psychological Medicine, Sydney Children’s Hospitals Network, Sydney, NSW 2031, Australia; 2 Discipline of Psychiatry and Mental Health, School of Clinical Medicine, Faculty of Medicine and Health, University of New South Wales, Sydney, NSW 2052, Australia; 3 Neuroinflammation Research Group, Centre for Immunology and Allergy Research, Westmead Institute for Medical Research, Sydney, NSW 2145, Australia; 4 School of Medical Sciences, Faculty of Medicine Health, The University of Sydney, Sydney, NSW 2050, Australia; 5 Dementia Research Centre, Macquarie Medical School, Faculty of Medicine, Health and Human Sciences, Macquarie University, Sydney, NSW 2109, Australia; 6 University of Kansas Alzheimer’s Disease Research Centre, Kansas City, KS 66160, USA; 7 Department of Biochemistry and Molecular Biology, University of Kansas Medical Centre, Kansas City, KS 66160, USA; 8 Department of Neurology, University of Kansas Medical Centre, Kansas City, KS 66160, USA; 9 Department of Psychiatry & Behavioral Sciences, University of California San Francisco, San Francisco, CA 94143, USA; 10 Department of Genetics, The University of Texas MD Anderson Cancer Center, Houston, TX 77030, USA; 11 Brain and Mind Centre, Translational Research Collective, Faculty of Medicine and Health, The University of Sydney, Sydney, NSW 2050, Australia; 12 Molecular Neurobiology Research Laboratory, Kids Research, Children’s Medical Research Institute, Children’s Hospital at Westmead, Westmead, NSW 2145, Australia; 13 Kids Neuroscience Centre, Kids Research, Children’s Hospital at Westmead, Westmead, NSW 2145, Australia; 14 Sydney Medical School, Faculty of Medicine and Health, The University of Sydney, Sydney, NSW 2050, Australia; 15 Murdoch Children’s Research Institute, Royal Children’s Hospital, Parkville, VIC 3052, Australia; 16 Department of Pediatrics, University of Melbourne, Parkville, VIC 3010, Australia; 17 Brain and Mind Centre, The University of Sydney, Sydney, NSW 2050, Australia; 18 Allied Health, Research and Strategic Partnerships, Nepean Blue Mountains Local Health District, Penrith, NSW 2750, Australia; 19 Centre for Transplant and Renal Research, Westmead Institute for Medical Research, Sydney, NSW 2145, Australia; 20 Renal and Transplant Medicine Unit, Westmead Hospital, Westmead, NSW 2145, Australia; 21 Westmead Clinical School, Faculty of Medicine and Health, The University of Sydney, Sydney, NSW 2050, Australia; 22 The Tumor Bank, Kids Research, Children’s Hospital at Westmead, Westmead, NSW 2145, Australia; 23 Children’s Cancer Research Institute, Children’s Hospital at Westmead, Westmead, NSW 2145, Australia; 24 Department of Molecular and Integrative Physiology, University of Kansas Medical Centre, Kansas City, KS 66160, USA; 25 Department of Immunopathology, Institute for Clinical Pathology and Medical Research-New South Wales Health Pathology, Sydney, NSW 2145, Australia

**Keywords:** transcriptomics, blood, biomarkers, machine learning, neurodegenerative diseases, Alzheimer’s disease

## Abstract

Alzheimer’s disease (AD) is a growing global health crisis affecting millions and incurring substantial economic costs. However, clinical diagnosis remains challenging, with misdiagnoses and underdiagnoses being prevalent. There is an increased focus on putative, blood-based biomarkers that may be useful for the diagnosis as well as early detection of AD. In the present study, we used an unbiased combination of machine learning and functional network analyses to identify blood gene biomarker candidates in AD. Using supervised machine learning, we also determined whether these candidates were indeed unique to AD or whether they were indicative of other neurodegenerative diseases, such as Parkinson’s disease (PD) and amyotrophic lateral sclerosis (ALS). Our analyses showed that genes involved in spliceosome assembly, RNA binding, transcription, protein synthesis, mitoribosomes, and NADH dehydrogenase were the best-performing genes for identifying AD patients relative to cognitively healthy controls. This transcriptomic signature, however, was not unique to AD, and subsequent machine learning showed that this signature could also predict PD and ALS relative to controls without neurodegenerative disease. Combined, our results suggest that mRNA from whole blood can indeed be used to screen for patients with neurodegeneration but may be less effective in diagnosing the specific neurodegenerative disease.

## 1. Introduction

Alzheimer’s disease (AD), the most common form of dementia, is a rapidly growing global medical crisis. With 50 million people currently affected and at least an additional 10 million cases a year predicted, the global cost to the economy is estimated to be 1.3 trillion USD annually [1]. The clinical diagnosis of AD has remained challenging, as 25–30% of patients are misdiagnosed with AD and a further 50–70% of patients with symptoms of AD do not receive a probable AD diagnosis from their primary care provider [2]. To combat this, there has been an increased focus on the identification of putative, blood-based biomarkers to aid in the early diagnosis and detection of AD. Diagnostic tests are especially important for identifying patients with AD who may be suitable for clinical trials [3]. The majority of this work has focused on the core pathological hallmarks of AD, including amyloid β (Aβ), phosphorylated tau (pTau), and neurofilament light chain (NfL) [4]. Although these markers have shown some diagnostic utility, for example, pTau181 [5], there is evidence that these biomarkers increase with age, even in the absence of clinical AD symptoms [4]. Further, modelling that has been done to demonstrate clinical efficacy of these tests has often been done on a smaller number of patients (often <100), cohorts with a significant class imbalance (healthy controls > AD), and rarely examine whether their biomarkers are also predictive of other neurodegenerative diseases. This may lead to significant bias and perhaps limit the clinical utility of these models [6,7]. Therefore, there is the imperative to evaluate the efficacy of additional AD biomarkers outside those traditionally used using modelling techniques that are more likely to be generalizable. 

Advances in high-throughput omic technologies have allowed the measurement of tens of thousands of genes and molecules that are dysregulated in disease states, making them novel tools for identifying biomarkers. However, historically, there has been a tendency to use these high-throughput techniques to test a priori hypotheses, potentially limiting the full appreciation of their diagnostic capacity. Further, these datasets are often analysed using arbitrary yet commonly used fold-change and corrected *p*-value thresholds, which can bias the results and their interpretation [8]. One way around these limitations is to distil new perspectives in high-throughput data, without preconceptions, using machine learning. Such an approach represents a unique and effective way to identify novel biomarkers. A small number of prior studies have used this approach to identify novel blood biomarker signatures in AD using transcriptomic and proteomic data [9,10,11,12,13,14,15]. Although these studies have shown promising results, there are some limitations that warrant consideration. First, many studies use a very low number of samples (sometimes <20) [10,11,12,13,15]. Small sample sizes in high-dimensional data, like patient-derived omic data, can result in significant problems in pattern recognition and model overfitting [16,17,18]. Furthermore, as mentioned previously, class imbalances where the number of samples in one group significantly outweighs that in the other group risk biasing the model in a particular direction, often toward the over-represented cohort. In many studies, the number of cognitively healthy controls far exceeds the number of AD patients and is reflected in high-specificity (ability to predict healthy controls) performance metrics [12]. Additionally, prior investigations utilizing large datasets from public repositories (e.g., Gene Omnibus (GEO) database), do not specify, either in the meta-data or the publication, the diagnostic criteria for AD [13,14,15,19], thereby limiting the generalizability of their findings to patients with AD.

There is substantial overlap in genetics, cellular pathways, and even clinicopathological features across neurodegenerative diseases [20,21,22,23]. Therefore, an important consideration in biomarker development is whether the signature(s) identified is specific to the disease of interest. Few machine learning studies for AD biomarker development have determined whether the identified signatures are similar or divergent in other neurodegenerative diseases. One study demonstrated that DNA methylome patterns in AD significantly overlap with those of Parkinson’s disease (PD) and amyotrophic lateral sclerosis (ALS) [24]. Two other research groups have reported that certain features (genes) selected by random forest algorithms overlap among neurodegenerative diseases, including AD, PD, ALS, frontotemporal dementia (FTD), Huntington’s disease (HD), and Friedrich’s Ataxia [15,19]. However, these studies relied on fold-change and *p*-value threshold cutoffs and only compared categories of affected transcripts rather than identifying whether their machine learning models themselves were able to predict other diseases relative to healthy controls. Thus, it remains unclear if AD predictive biomarker models are useful for diagnosis or whether their signature overlaps with those of other neurodegenerative diseases.

To address this knowledge gap, we analysed transcriptomic microarray data from whole-blood samples of clinically diagnosed AD patients and healthy controls. To move away from fold-change- and *p*-value-based identification of dysregulated genes, we used an unbiased combination of unsupervised machine learning and functional enrichment analyses to identify these genes. We then used random forest models to test whether our identified gene biomarker candidates were indeed specific to AD or, using the same models, whether they were generalizable to two other neurodegenerative diseases: PD and ALS. 

## 2. Results

### 2.1. Characteristics of the Included Datasets

Our review of the GEO database identified five whole-blood microarray datasets that met the inclusion criteria: GSE140829, GSE97760, GSE85426, GSE63061, and GSE63060. Two of these, GSE140829 and GSE85426, did not specify the criteria used to diagnose probable AD and were thus excluded from our analyses. Samples from the included datasets consisted of patients with a diagnosis of possible or probable AD and cognitively normal controls at the time of assessment (Table 1). Donor age (ranging from 72 to 79 years old) and sex were generally well matched, both across and within datasets (Table 1). Only one of the three datasets specified the ethnicity of their donors: GSE97760 was made up of primarily white donors with two AD donors who were African American [25]. Although all the datasets reported measuring RNA integrity (RIN), none reported the cutoff used. Further, none of the datasets reported the APOE genotype of the donors.

### 2.2. Principal Component and Functional Network Analyses of GSE97760 Indicate Dysregulated Pathways and Central Gene Nodes in Whole Blood of AD Patients

**Figure 1:**
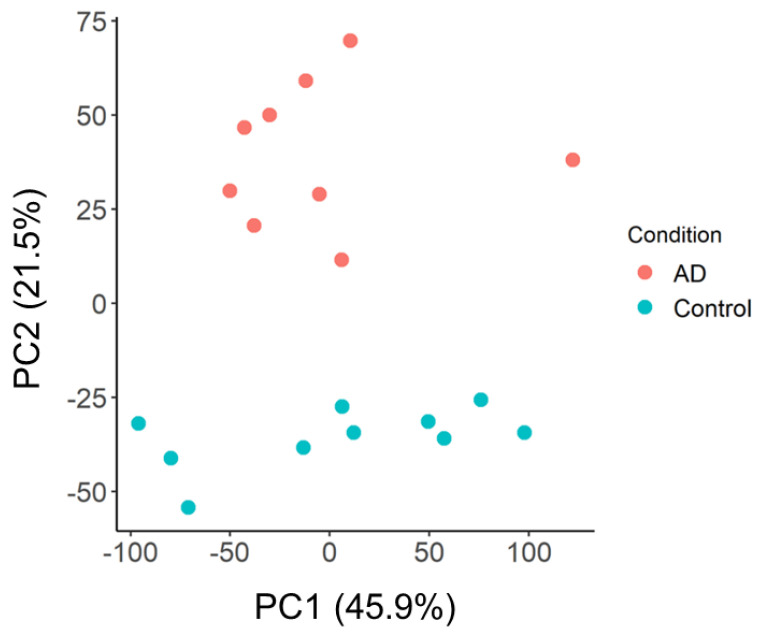
Principal component analysis (PCA) of AD reference dataset, GSE97760.

We first performed feature selection using principal component analysis (PCA) on GSE97760 to identify dysregulated central gene nodes that may predict an AD diagnosis. GSE97760 was selected as a reference dataset, as all its AD donors had been clinically diagnosed as having advanced AD [25]. PCA showed that there was a distinct separation between the AD and control samples (Figure 1). 

It was also clear that the groups were separated along the y-axis, suggesting that genes within PC2 played a key role in driving group separation. Given that PC1, however, contributed to a greater percentage of the variance between groups (45.9% vs. 21.5%), we also wanted to ensure that we examined the possibility that genes in PC1 may be good predictors of AD. We, therefore, took the top 1000 genes that correlated with PC1 (Supplementary Table S1) and PC2 (Supplementary Table S2), respectively, as the most dysregulated genes between AD and controls. 

**Figure 2:**
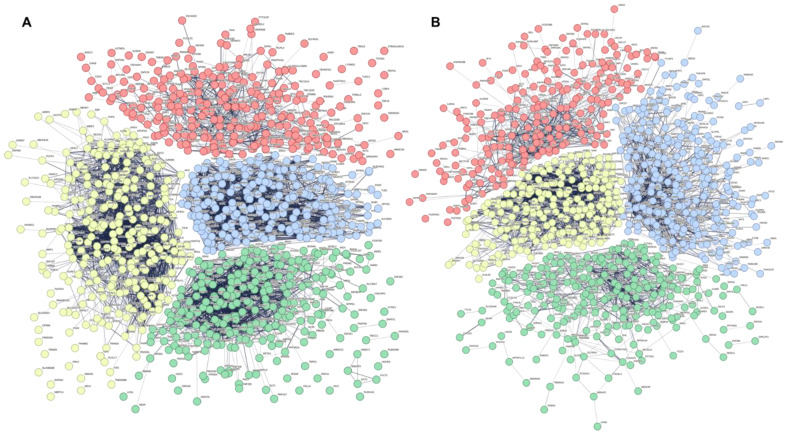
K-means clustering of top 1000 dysregulated genes in GSE97760 identified using principal component analysis. (**A**) Principal component (PC) 1. Red n = 255, yellow n = 271, green n = 250, and blue n = 224 genes. (**B**) PC2. Red n = 239, yellow n = 216, green n = 261, and blue n = 284 genes. Gene names are listed in Supplementary Tables S1 and S2.

To identify the number of common pathways and interconnections represented in these genes, we performed k-means clustering in STRING [26]. Four clusters were identified as being the optimal number for the dysregulated genes in PC1 and PC2, respectively (Figure 2). 

Genes within each k-means cluster were then independently examined, again using STRING, to identify overlapping pathways and central gene nodes (the genes that are the most connected within the network) within the cluster. The pathways’ biological function and cellular localization were both characterized using Gene Ontology (GO). In PC1, the first k-means cluster (red) was characterized by genes involved in cellular localization within the endomembrane system (Supplementary Figure S1). There were three central gene nodes identified as playing a role in vesicle formation, the SNARE complex, and signal transduction (Table 2; Supplementary Table S3). The second k-means cluster (yellow) was characterized by 62 central gene nodes involved in metabolic processes across the mitochondrion and ribonucleoprotein complex and included genes from ATP synthase (complex V), mitochondrial ribosomes, and those with roles in mitochondrial respiration (Table 2; Supplementary Table S4; Supplementary Figure S2). The third k-means cluster (green) represented genes important for gene expression in the nucleus (Supplementary Figure S3). The 39 central gene nodes identified here were involved in RNA regulation and transcription (Table 2; Supplementary Table S5). The fourth and final k-means cluster (blue) from PC1 included genes involved in cellular response to stimulus and protein folding, both of which localize to the cytosol (Supplementary Table S6). There were 23 central gene nodes involved in protein folding, maintenance, stabilization, and degradation (Table 2; Supplementary Figure S4).

We observed a degree of overlap in the biological function of genes between PC1 and PC2 k-means clusters. The first PC2 k-means cluster (red) was similarly characterized by gene expression in the nucleus; however, unlike PC1, it also included genes expressed in the ribonucleoprotein complex (Supplementary Table S7). Here, the 16 central gene nodes played roles in mRNA regulation and mitochondrial ribosomes (Table 3; Supplementary Figure S5). Genes involved in metabolic processes were also identified in the second PC2 k-means cluster (yellow). These were in both the cytosol and mitochondrion and included 41 central gene nodes involved in cytochrome c oxidase (complex IV), mitochondrial ribosomes, NADH dehydrogenase (complex I), and ribosomes (Table 3; Supplementary Table S8; Supplementary Figure S6). The final two k-means clusters represented pathways unique to PC2. The third k-means cluster (green) included 18 central gene nodes involved in transport within the cytoplasm, including vesicular transport, protein trafficking, and haemoglobin (Table 3; Supplementary Table S9; Supplementary Figure S7). The final k-means cluster (blue) was made up of 12 central gene nodes involved in the regulation of cellular processes in the plasma membrane (Supplementary Figure S8). These included genes with roles in protein kinase signalling, estrogen signalling, transcription, and protein chaperones (Table 3; Supplementary Table S10).

### 2.3. Supervised Machine Learning Identifies Dysregulated Pathways That Can Predict AD

**Figure 3:**
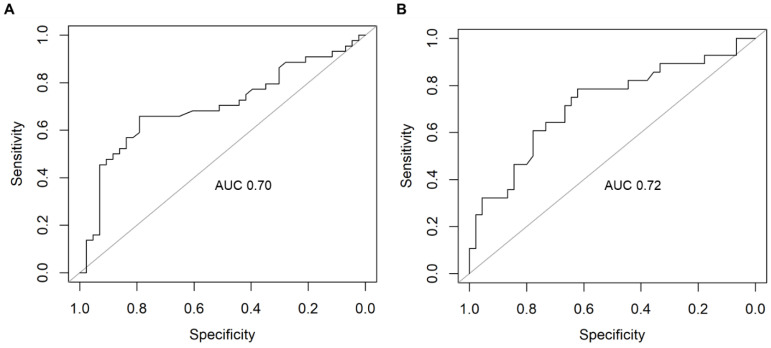
Receiver operating characteristic (ROC) curve of the random forest model’s performance for PC1 gene expression cluster in (**A**) dataset A, GSE63061, and (**B**) dataset B, GSE63060. AUC: area under the curve.

We used supervised machine learning (random forest) to determine which clusters were the best predictors of AD. Importantly, this was performed separately for GSE63061 (dataset A) and GSE63060 (dataset B) to assess the reproducibility and generalizability of our model’s performance. For PC1, the top-performing cluster was gene expression, demonstrating consistently higher sensitivity and precision, with averages of 0.67 and 0.77, respectively (Table 4; Figure 3).

**Figure 4:**
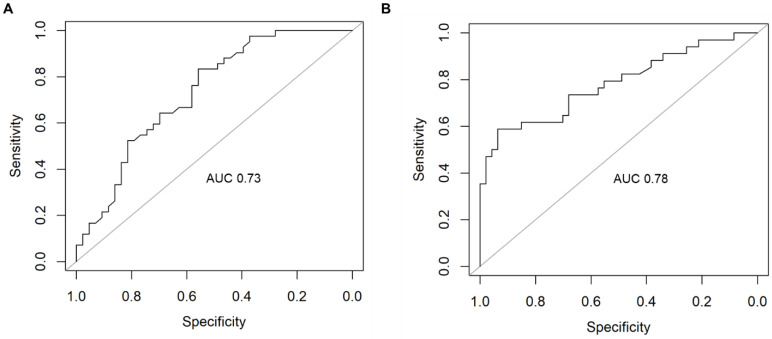
Receiver operating characteristic (ROC) curve of the random forest model’s performance for PC2 metabolic process cluster in (**A**) dataset A, GSE63061, and (**B**) dataset B, GSE63060. AUC: area under the curve.

For PC2, the top-performing cluster was metabolic process, with average sensitivity of 0.7 and precision of 0.77 (Table 5; Figure 4).

### 2.4. Feature Selection of the Top-Performing Genes That Contribute to AD Prediction within Each PC Cluster

**Figure 5:**
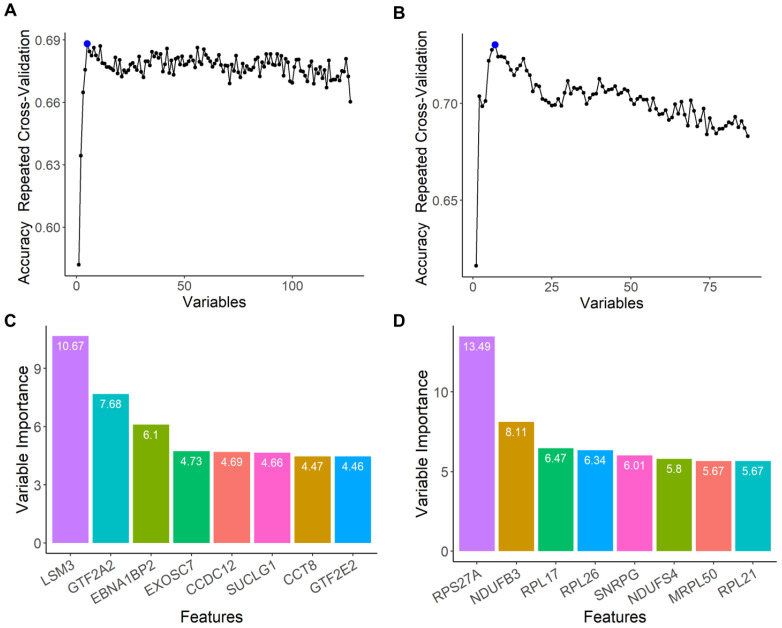
Feature selection of the top-performing central gene nodes using recursive feature elimination (RFE) for (**A**) PC1, gene expression cluster, and (**B**) PC2, metabolic process cluster. The blue dot indicates peak performance of the model where features were identified from. (**C**,**D**) The eight top-performing genes for the (**C**) PC1 gene expression cluster and (**D**) PC2 metabolic process cluster.

We next sought to identify the top-performing gene within each PC cluster that contributed to the random forest model’s ability to predict AD. Recursive feature elimination (RFE) was performed on the PC1 gene expression cluster (Figure 5A) and the PC2 metabolic process cluster (Figure 5B), respectively. At the model’s top performance (blue dot, Figure 5A,B), one gene from each cluster dominated the predictive power: *LSM3* (PC1 gene expression; Figure 5C), a component of the U4/U6-U5 tri-snRNP complex involved in pre-mRNA splicing and spliceosome assembly, and *RPS27A* (PC2 metabolic process; Figure 5D), a component of the 40S subunit of the ribosome that plays a role in protein synthesis. The eight top-performing genes for the PC1 gene expression cluster included those involved in spliceosome assembly, RNA binding, and transcription (Table 6). On the other hand, the top performers for the PC2 metabolic process cluster included genes involved in protein synthesis, mitoribosomes, and NADH dehydrogenase (Table 6).

### 2.5. Genes That Predict AD Are Also Predictive of Neurodegenerative Diseases

When identifying new potential biomarkers, it is important to determine if they are disease-specific (i.e., AD). We, therefore, sought to test whether our top-performing gene candidates for AD were also predictive of Parkinson’s disease (PD) and amyotrophic lateral sclerosis (ALS), among the most common neurodegenerative diseases. More specifically, we wanted to determine whether our top-performing genes were also able to predict PD and ALS patients irrespective of whether they were trained using an AD dataset (AD training set and PD or ALS test set) or disease-specific dataset (PD or ALS training set and PD or ALS test set). For Parkinson’s disease, we sourced two microarray datasets from GEO, GSE6613 (PD vs. healthy control) and GSE72267 (drug-naïve PD vs. healthy control) (Table 7). We also identified one ALS microarray dataset, GSE112681 (ALS vs. healthy control) (Table 7). 

Random forest models for the top AD gene performers from the PC1 gene expression cluster and PC2 metabolic process cluster, respectively, were trained using a collapsed AD dataset made up of samples from GSE63061 and GSE63060. The trained models were then tested on each of the three PD and ALS datasets, GSE6613, GSE72267, and GSE112681. The random forest models for the PC1 gene expression cluster had precision metrics of >0.65, suggesting that they were able to identify true PD and ALS cases (Table 8).

The models for the PC2 metabolic process cluster had precision metrics of >0.68 and went as high as 0.83, similarly demonstrating that these genes were predictive of PD and ALS (Table 9).

It is worth noting that while all the models for the PC1 gene expression and PC2 metabolic process clusters had good predictive value for neurodegenerative disease patients (indicated by high sensitivity and precision), they were unable to identify the healthy controls in each dataset (indicated by low specificity and AUC).

To further validate these findings, we tested our models’ performance when both trained and tested on the same disease dataset (70% for training, 30% withheld for testing). This improved all performance metrics for both the PC1 gene expression cluster (Table 10) and PC2 metabolic process cluster (Table 11). Furthermore, it also improved the ability of the models to accurately identify healthy controls (higher specificity) (Table 10 and Table 11). 

## 3. Discussion

An affirmative diagnosis for neurodegenerative diseases remains difficult and elusive. Currently, there is a reliance on neuroimaging and measurements of biomarkers in cerebrospinal fluid (CSF) [27]. While these do provide clinical utility, there are caveats, namely, accessibility to neuroimaging (particularly in lower-socioeconomic countries) and the perceived invasiveness of CSF collection [27,28]. Further, targeted proteomic or metabolomic methods for analysing CSF are costly and methodologically challenging [27]. PCR-based approaches to examine mRNA changes are a favourable alternative, given that these assays are timely, reliable, robust, relatively simple, and cost-efficient [29]. However, putative biomarker measurements employing this approach are still in their infancy, hence the need to explore the possibility of reliable blood mRNA biomarkers. Here, we examined publicly available microarray data using machine learning to determine if whole-blood transcriptomic signatures are unique to AD or whether they are reflective of other neurodegenerative diseases, including PD and ALS. Our results suggest that mRNA from whole blood can indeed be used to screen for patients with neurodegeneration but may be less effective in diagnosing the specific disease. 

Our unsupervised machine learning (PCA and k-means clustering) and functional enrichment analyses indicated that there are multiple dysregulated pathways and central gene nodes in the blood of AD patients. Dysfunctional metabolic processes in the mitochondria, cytosol, and ribonucleoprotein complex were found across both PCs, highlighting that these processes are likely dysregulated in the periphery of neurodegenerative disease patients. Central gene nodes involved in these processes include those for NADH dehydrogenase (complex I), cytochrome oxidase C (complex IV), and ATP synthase (complex V), as well as those involved in mitoribosomes and mitochondrial respiration. Similarly, gene expression was found to be dysregulated across the nucleus and ribonucleoprotein complex, implicating processes such as RNA regulation, transcription, and mitoribosome function. Unsurprisingly, both gene expression and metabolic process central gene nodes were found to be the top-performing clusters across PC1 and PC2, respectively, showing acceptable levels of sensitivity and precision. Additional feature selection using RFE identified that sixteen (eight from the PC1 gene expression cluster and eight from the PC2 metabolic process cluster) central gene nodes drove the models’ predictive performance. These included those involved in spliceosome assembly, RNA binding, transcription, protein synthesis, mitoribosomes, and NADH dehydrogenase. Despite the AD transcriptomic signatures identified, subsequent machine learning demonstrated that these were not unique to AD. Our models using these top-performing genes were also able to predict PD and ALS patients irrespective of whether they were trained using an AD dataset (AD training set and PD or ALS test set) or disease-specific dataset (PD or ALS training set and PD or ALS test set). Importantly, the medications commonly used to treat the symptoms of AD, PD, and ALS are different and the ability of our models to perform across neurodegenerative diseases suggests that our findings are not simply an artefact of drug treatments. It is worthy to note, however, that only one of the datasets specified drugs used by the donors: the drug-naïve PD dataset [30]. Although our models showed strong metrics, this dataset was small (n = 60). Future research, therefore, should include current prescription and non-prescription data of their donors to ensure that these can be controlled for as well as larger numbers to enable more generalizable conclusions. 

Many of the genes that we found to be good predictors of AD, PD, and ALS have also been previously identified as being broadly implicated in neurodegeneration. *RPS27A* has been linked to mild cognitive impairment (MCI) and AD [31,32]. It has also been shown to interact with tau and lead to microglial activation that triggers subsequent widespread neurodegeneration [33,34]. Both *MRPL50* and *NDUFB3* have been implicated in AD, glaucoma, and age-related neurodegeneration [35,36]. In addition to being identified as a gene that links MCI progressing to AD [31], *LSM3* has been implicated in PD [37], AD [36], the adult-onset neurodegenerative disorder Fragile X Tremor and Ataxia syndrome [38]. *SUCLG1* is decreased in AD brains [36], and mutations in this gene have been linked to encephalomyopathic mitochondrial DNA (mtDNA) depletion syndromes characterized by hypotonia and pronounced neurological symptoms [39,40]. Interestingly, mtDNA is well documented to play a role across neurodegenerative diseases, including but not limited to AD, PD, and ALS [41,42], suggesting that *SUCLG1* may be an interesting gene to further look at in the context of neurodegeneration. Further, we implicated *NDUFS4* in our neurodegeneration predictive models, which has been connected to both AD [36] and in neurodegeneration associated with the mitochondrial disorder Leigh Syndrome [43,44]. To our knowledge, the predictive genes identified are not described as genetic risk factors for neurodegenerative diseases by any Genome-Wide Association Study (GWAS). Future studies may benefit from identifying if there are any mutations in these genes that are disease-associated.

While we provide strong evidence that mRNA signatures can be used to indicate whether neurodegeneration is present, there are some limitations to this work. First, we used a specific feature selection method (unsupervised machine learning and functional enrichment analyses) to narrow down a list of >15,000 genes to only 16. Therefore, it may be the case that we have overlooked other genes and genetic signature(s) with greater sensitivity towards AD or other neurodegenerative diseases. However, this may have been mitigated using our selection approach. Our PCA identified the genes highly correlated with the principal components—i.e., those with the greatest influence on driving group separation between AD patients and healthy controls. Inherent to this is that the non-identified genes contribute less to this differentiation and are, therefore, highly likely to be poor predictors of AD. Further, our deliberate exclusion of *p*-value and fold-change thresholds ensured that our results were not biased toward arbitrary cutoffs. The use of functional enrichment analyses to uncover central gene nodes (the genes that are the most connected within the network) should enrich for genes whose dysregulation has the highest disruptive potential for the system or pathway. In doing so, we may have also inadvertently examined genes that are functionally connected to these central gene nodes. In this systems biology approach, dysregulation of a central, highly connected gene is also likely to disrupt downstream connections. Despite this, it can be argued that our functional enrichment analysis is biased toward what is already known and that genes with a subtle influence may be unique to neurodegenerative diseases. These subtleties are unlikely to be uncovered using tools like databases and machine learning; future research could, therefore, benefit from identifying other methods to examine them. Further, due to the small sample numbers in the PD and ALS datasets, we were unable to parse these into feature selection, training, and test sub-datasets. It may be the case, therefore, that some genes predictive of PD and ALS may not be predictive of AD. Future studies should aim to generate larger transcriptomic datasets, which would enable our analytical method to be used on PD and ALS samples.

Another potential limitation of this work is that our models generally had low specificity and high precision, indicating that the models performed poorly in identifying true negatives (people without neurodegeneration). In the AD datasets, all healthy controls were in their early to mid-70s and may, therefore, have demonstrated subclinical, age-related, non-pathological neurodegeneration [45]. This conclusion is strengthened by two findings from our data. First, our models’ sensitivity and precision metrics were higher for the AD dataset GSE63060 relative to GSE63061, where the age of controls was 72 compared with 75, respectively. In the future, research could benefit from testing whether blood-based biomarkers lose sensitivity and precision with the increase in patient age. Second, when our models were trained on the AD datasets and then tested on the PD or ALS datasets, whose healthy controls were around 10 years younger, the specificity was very low. When we instead trained and tested the models on the same dataset (PD or ALS, respectively), the specificity improved dramatically. This also suggests that our models are readily able to differentiate between a patient with neurodegeneration and a healthy control who is younger.

It is also important to consider that there are likely to be sex differences in neurodegenerative disease pathogenesis and thus biomarkers [46]. For example, a recent study demonstrated that plasma phospho-tau threonine 217 (p-Tau217) and NfL levels differed between males and females with autosomal dominant AD [47]. From a diagnostic testing perspective, however, it is important to identify sex-independent biomarkers that can be routinely used in the clinic. In the present study, although we used a female-only AD dataset as our reference to identify dysregulated central gene nodes, our models still showed good performance (sensitivity and precision) when trained and tested using AD, PD, and ALS datasets made up of both males and females. This suggests that although we used females as a reference point, we identified genes that are less likely to be sex-specific and may thus be clinically useful to identify patients with neurodegeneration. 

The final limitation of this work is that the datasets used in the present study are not derived from single-cell RNA sequencing, which may limit our interpretation of results. Whole blood is made up of many cell types, including red blood cells, white blood cells (lymphocytes, monocytes, and granulocytes), and platelets. Mature red blood cells are enucleated and thus have low levels of mRNA, with estimates that only about 10% contain mRNA [48,49]. This suggests that the bulk of mRNA comes from the various white blood cells or platelets [50,51], highlighting that there may be peripheral dysfunction in these diseases driven by these cell types. For example, CD49^+^ Tregs, relative to other immune cell subsets, have been shown to be increased in blood samples of PD patients relative to healthy controls [52]. This suggests that the relative proportion of blood cell types in a sample may influence the identification of disease-related changes in gene expression, potentially limiting clinical utility [51]. This may especially be the case in patients with comorbid conditions requiring immunosuppression, like cancer patients undergoing chemotherapy. A recent study, however, demonstrated that there were marked differences in CD4^+^ memory, CD4^+^ activated, and CD8^+^ naïve cells, as well as CD38^+^CD16^low^ monocytes, between AD and PD patients [53]. Given that our models were reasonable across AD and PD, as well as ALS, the relative abundance of cell subsets may not provide diagnostic utility with respect to neurodegeneration. Additionally, it is important to note that we do not know if our dysregulated genes are from the CNS or only the periphery. Extracellular vesicles (EVs) package mRNA and may cross the blood–brain barrier into general circulation. There is a poor understanding, however, of how EVs achieve this in a bidirectional manner (i.e., cross from brain to blood and vice versa) [54,55]; therefore, we cannot conclude the relative proportion of EVs from these data. Future studies could greatly benefit from dissecting this further to determine whether these blood-based genetic changes are driven by immune cell subtypes or whether they are cell-subtype-independent. 

In summary, we report a machine learning and functional enrichment analysis approach to identifying whole-blood transcriptomic signatures in AD. We also investigated whether these transcriptomic signatures were unique to AD or whether they were indicative of neurodegenerative disease more broadly. The top-performing genes for predicting AD included those involved in spliceosome assembly, RNA binding, transcription, protein synthesis, mitoribosomes, and NADH dehydrogenase. Although we did identify a blood-based transcriptomic signature that was predictive of AD, we found that it could also be used to identify PD or ALS patients relative to non-neurodegenerative disease controls. Together, these findings suggest that there is a shared blood molecular signature across neurodegenerative diseases. This highlights that mRNA from whole blood can likely be used to screen patients for neurodegeneration but is less effective in diagnosing the specific neurodegenerative disease. Our identified gene signature should be experimentally investigated to determine whether it is indeed a viable clinical screening test to identify neurodegenerative diseases in patients.

## 4. Materials and Methods

### 4.1. Identification of Publicly Available Transcriptomic Datasets

We identified publicly available transcriptomic datasets using a systemic search of the Gene Expression Omnibus (GEO) database. The key term used for the search was “Alzheimer’s disease”, and the results were limited to homo sapiens. Datasets were included on the basis that they (a) examined gene expression in whole-blood samples, (b) used a microarray to generate high-throughput transcriptomic data, (c) clinically confirmed AD diagnosis, and (d) included cognitively normal healthy controls. We excluded datasets generated using RNA sequencing due to their small sample sizes, which increases the risk of developing overfitted, ungeneralizable models using our machine learning methods. Three AD datasets were included: GSE97760, GSE63061, and GSE63060. 

### 4.2. Data Processing

After downloading the identified GEO datasets, we first confirmed that the data were pre-normalized and did not need additional normalization. Log transformations were not used, as they are not required in the absence of traditional statistical and fold-change analyses. The three datasets were processed independently; duplicate gene entries were removed; a z-score was calculated for each gene. A list of genes identified in each dataset was generated, and genes not common to all three datasets were discarded. Data processing, merging, and PCA were performed in R Studio v1.2.5033 (R v3.6.3) using GEOquery, dplyr, and PCA, and visualization was performed using ggplot. 

### 4.3. Feature Selection Using Unsupervised Machine Learning Methods and Functional Enrichment Analyses on GSE97760

**Figure 6:**
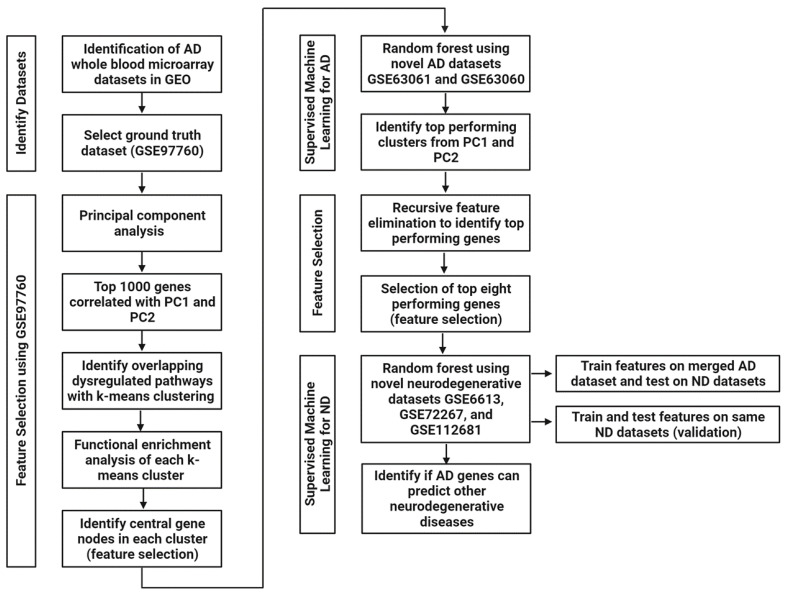
Flow chart of the methodological pipeline. Abbreviations: PC: principal component; ND: neurodegenerative diseases.

We first examined the three AD datasets and selected a ground-truth dataset, GSE97760. This was selected due to all the patients having a diagnosis of advanced probable AD, thereby reducing the chances of clinical misdiagnosis [25]. Further supporting this, a PCA indicated a clear group separation between the AD cases and healthy controls (Figure 1). Using GSE97760, we then performed our established two-stage machine learning pipeline as previously described in Finney et al. [8]. Briefly, we first analysed all genes (>15,000) using PCA (Figure 6). Importantly, PCA allows us to reduce dimensionality and minimize potential information loss [56] while simultaneously revealing hidden patterns in high-throughput transcriptomic data [57]. PCA was performed in R Studio v1.2.5033 (R v3.6.3) and visualized using ggplot. The top 1000 genes correlating with PC1 and PC2 were identified and selected as potential biomarker gene candidates (Figure 6). The top 1000 genes were then entered into STRING v11 [26,58] for gene enrichment analysis and to identify interaction networks. We first performed unsupervised machine learning using k-means clustering to identify the presence of overlapping functional network clusters in the genes (Figure 6). Each k-means cluster was then independently entered into STRING for network analyses (Figure 6). The following active interaction sources were used: experiments, databases, co-expression, neighbourhood, and gene fusion. The minimum interaction score was set to 0.7 (high confidence). The clusters were then characterized by biological processes and cellular localization using Gene Ontology (GO) [59], and the central gene nodes in each cluster were selected. Central gene nodes were identified as those with the highest number of connections with other genes in the network, a method used to increase the likelihood of identifying biomarker candidates that are fundamental to biological processes [8]. 

### 4.4. Supervised Machine Learning to Identify the Best Predictors of AD

Once the central gene nodes from each k-means cluster were identified, we applied supervised machine learning (random forest) to determine which central gene nodes (features) were best able to distinguish AD patients from cognitively healthy controls. To do so, we used two novel AD datasets to train and test the models: GSE63061 and GSE63060. Both datasets, respectively, were split into training (70%) and test (30%) datasets. The training datasets were used to perform model training, tuning, and validation. A 5-fold cross-validation repeated three times was used to improve model accuracy and identify the top-performing gene biomarker predictors [60]. The final evaluation of our random forest models was performed on the withheld test datasets. Performance indicators used to evaluate our models included sensitivity (correctly identifies AD patients) and precision (quality of positive AD prediction, i.e., number of AD patients/total number of predicted AD patients (true and false)). Using these metrics to determine the diagnostic utility of biomarker tests is particularly important because (a) it reduces the likelihood of producing a false-negative outcome (someone who does have AD is identified as being healthy) and (b) assesses the probability that a person with a positive result indeed has AD [61]. We did, however, also report additional performance metrics, including AUC (ability to distinguish between AD patients and cognitively healthy controls) and specificity (correctly identified healthy controls). Random forest modelling was performed in R Studio v1.2.5033 using the libraries rpart, caret, and pROC. 

### 4.5. Feature Selection to Identify the Genes That Contribute to AD Prediction 

Within each of the best predictive models for the PC1 and PC2 clusters, respectively, we wanted to identify the genes that contributed the most to our models’ ability to predict AD cases. To do so, we performed RF-RFE and examined variable importance at the peak performance of each model (Figure 6). This method has previously been used to successfully identify important genes in transcriptomic data [62]. We then took the top eight important genes from each model to use as features in our neurodegenerative disease models (Figure 6). 

### 4.6. Supervised Machine Learning to Identify if AD Gene Biomarkers Are Unique to AD or Generalizable to Other Neurodegenerative Diseases

To identify whether our AD blood-based gene biomarkers were specific to AD, we tested whether the top eight important genes from each of the PC1 and PC2 clusters, respectively, were predictive of PD and ALS. We first identified three additional datasets in the GEO database that used a microarray to analyse whole blood from patients with PD (GSE6613 and GSE72267) and ALS (GSE112681). Importantly, we did identify datasets examining other dementias, including behavioural variant frontotemporal dementia, and there was a substantial class imbalance whereby the number of healthy controls significantly outweighed the number of patients. Our modelling methods are not possible with such imbalanced datasets, and these were thus excluded from our analyses. These datasets were processed in the same way as the AD datasets described above in Section 4.2. 

We then used the eight top important genes from PC1 and PC2 clusters in AD in a random forest model (Figure 6). We first merged the two AD datasets (GSE63061 and GSE63060) into a single dataset using z-scores as we have previously done [8]. This merged dataset was then used as a training dataset to train, tune, and validate our models. A 5-fold cross-validation repeated three times was also used. We then used each of the three neurodegenerative disease datasets, respectively, to test these AD-trained models (Figure 6). We also conducted a second validation experiment where our random forest models were both trained and tested on each of the neurodegenerative disease datasets (Figure 6). Each dataset was split into a 70% training, fine-tuning, and validation dataset and a 30% withheld dataset for testing the models. As before, performance indicators used to evaluate our models included sensitivity (correctly identifies PD or ALS patients) and precision (quality of positive PD or ALS prediction, i.e., number of PD or ALS patients/total number of predicted PD or ALS patients (true and false)).

## Figures and Tables

**Table 1:** Characteristics of the included AD datasets.

	GSE97760		GSE63061		GSE63060	
	AD	Control	AD	Control	AD	Control
Sample size	9	10	139	134	145	104
Sex (M:F)	0:9	0:10	54:85	53:81	46:99	42:62
Age (average ± SEM)	79.3 ± 4.1	72.1 ± 4.1	77.9 ± 0.6	75.3 ± 0.5	75.4 ± 0.6	72.4 ± 0.6
AD diagnostic criteria	NIH diagnostic guidelines for AD		NINCDS-ADRDA and DSM-IV		NINCDS-ADRDA and DSM-IV	
Platform	GPL16699		GPL10558		GPL6947	

Abbreviations: DSM-IV: Diagnostic and Statistical Manual of Mental Disorders; NIH: National Institutes of Health; NINCDS-ADRDA: National Institute of Neurological and Communicative Disease and Stroke and Alzheimer’s disease.

**Table 2:** Characterization summary of each k-means cluster from PC1.

k-Means Cluster	Biological Function (GO)	FDR	Cellular Component (GO)	FDR	Number of Central Gene Nodes	Roles of Central Gene Nodes
1 (Red)	Cellular localization	3.61 × 10^−11^	Endomembrane system	6.02 × 10^−22^	3	Vesicle formation; SNARE complex; signal transduction
2 (Yellow)	Metabolic process	5.29 × 10^−25^	Mitochondrion Ribonucleoprotein complex	2.87 × 10^−40^ 5.93 × 10^−12^	62	ATP synthase (complex V); mitochondrial ribosomes; mitochondrial respiration
3 (Green)	Gene expression	3.87 × 10^−28^	Nucleus	1.75 × 10^−44^	39	RNA regulation; transcription
4 (Blue)	Cellular response to stimulus Protein folding	1.69 × 10^−9^ 1.19 × 10^−16^	Cytosol	2.8 × 10^−9^	23	Protein folding, maintenance, stabilization, and degradation

Abbreviations. FDR: false-discovery rate; GO: Gene Ontology.

**Table 3:** Characterization summary of each k-means cluster from PC2.

k-Means Cluster	Biological Function (GO)	FDR	Cellular Component (GO)	FDR	Number of Central Gene Nodes	Roles of Central Gene Nodes
1 (Red)	Gene expression	2.32 × 10^−9^	Nucleus Ribonucleoprotein complex	1.83 × 10^−21^ 0.00024	16	mRNA regulation; mitochondrial ribosomes
2 (Yellow)	Metabolic process	2.58 × 10^−22^	Cytosol Mitochondrion	1.78 × 10^−16^ 4.40 × 10^−6^	41	Cytochrome c oxidase (complex IV); mitochondrial ribosomes; NADH dehydrogenase (complex I); ribosomes
3 (Green)	Transport	4.58 × 10^−6^	Cytoplasm	4.19 × 10^−10^	18	Vesicular transport; protein trafficking; haemoglobin
4 (Blue)	Regulation of cellular processes	2.49 × 10^−10^	Plasma membrane	0.00062	12	Protein kinase signalling; estrogen signalling; transcription; protein chaperone

Abbreviations. FDR: false-discovery rate; GO: Gene Ontology.

**Table 4:** Random forest performance metrics for the dysregulated clusters from AD PC1.

	AUC		Sensitivity		Specificity		Precision	
Cluster	A	B	A	B	A	B	A	B
Cellular localization	0.53	0.54	0.48	0.64	0.39	0.26	0.31	0.67
Metabolic process	0.55	0.79	0.54	0.72	0.46	0.81	0.51	0.89
Gene expression *	0.70	0.72	0.65	0.69	0.66	0.61	0.65	0.88
Cellular response to stimulus/protein folding	0.64	0.74	0.58	0.68	0.59	0.69	0.55	0.89

* indicates top-performing cluster. Dataset A: GSE63061; dataset B: GSE63060.

**Table 5:** Random forest performance metrics for the dysregulated clusters from AD PC2.

	AUC		Sensitivity		Specificity		Precision	
Cluster	A	B	A	B	A	B	A	B
Gene expression	0.68	0.64	0.73	0.65	0.53	0.58	0.56	0.78
Metabolic process *	0.73	0.78	0.65	0.75	0.67	0.72	0.7	0.83
Transport	0.58	0.43	0.55	0.60	0.48	0.55	0.55	0.87
Regulation of cellular process	0.57	0.58	0.54	0.67	0.42	0.41	0.40	0.64

* indicates top-performing cluster. Dataset A: GSE63061; dataset B: GSE63060.

**Table 6:** Biological function of the top-performing genes for PC1 gene expression cluster and PC2 metabolic process cluster.

Abbreviation	Name	Function (NCBI)
PC1 Gene Expression Cluster		
*LSM3*	U6 snRNA and mRNA Degradation Associated	Component of the U4/U6-U5 tri-snRNP complex involved in pre-mRNA splicing and spliceosome assembly
*GFT2A2*	General transcription factor IIA subunit 2	Component of RNA polymerase II transcription machinery, role in transcriptional activation
*EBNA1BP2*	EBNA1 binding protein 2	RNA binding activity
*EXOSC7*	Exosome component 7	Enables RNA binding, 3′-5′-exoribonuclease activity, and RNA metabolism
*CCDC12*	Coiled-coil domain containing 12	Component of U2-type and post-mRNA release spliceosomal complexes
*SUCLG1*	Succinate-CoA ligase GDP/ADP-forming subunit alpha	Alpha subunit of the heterodimeric enzyme succinate coenzyme A ligase, catalyses conversion of succinyl CoA and ADP to succinate and ATP or of GDP to GTP
*CCT8*	Chaperone containing TCP1 subunit 8	Component of molecular chaperonin-containing T-complex (TRiC), assists in folding of proteins upon ATP hydrolysis
*GTF2E2*	General transcription factor IIE subunit 2	Component of RNA polymerase II transcription initiation complex, involved in recruitment of general transcription factor IIH to initiation complex and stimulation of RNA polymerase II C-terminal domain kinase and DNA-dependent ATPase activity
PC2 Metabolic Process Cluster		
*RPS27A*	Ribosomal protein 27A	Component of 40S subunit of the ribosome involved in protein synthesis
*NDUFB3*	NADH:ubiquinone oxidoreductase subunit B3	Component of accessory subunit of mitochondrial membrane respiratory chain NADH dehydrogenase (complex I)
*RPL17*	Ribosomal protein L17	Component of the ribosomal 60S subunit, involved in protein synthesis
*RPL26*	Ribosomal protein L26	Component of the ribosomal 60S subunit, involved in protein synthesis
*SNRPG*	Small nuclear ribonucleoprotein polypeptide G	Component of the U1, U2, U4, and U5 small nuclear ribonucleoprotein complexes, involved in processing of 3′ end of histone transcripts
*NDUFS4*	NADH:ubiquinone oxidoreductase subunit S4	Component of nuclear-encoded accessory subunit of mitochondrial membrane respiratory chain NADH dehydrogenase (complex I)
*MRPL50*	Mitochondrial ribosomal protein L50	Encodes a putative 39S subunit protein of mitochondrial ribosomes (mitoribosomes)
*RPL21*	Ribosomal protein L21	Component of the ribosomal 60S subunit, involved in protein synthesis

**Table 7:** Characteristics of the PD and ALS datasets.

	GSE6613		GSE72267		GSE112681	
	PD	Control	PD	Control	ALS	Control
Sample size	50	22	40	20	167	137
Sex (M:F)	39:11	11:11	22:18	10:10	96:68	79:58
Age (average ± SEM)	69.4 ± 1.2	64.4 ± 2.3	68.8 ± 1.1	60.3 ± 1.3	Not reported	Not reported
Diagnostic criteria	United Kingdom Parkinson’s Disease Society Brain Bank clinical diagnostic criteria		United Kingdom Parkinson’s Disease Society Brain Bank clinical diagnostic criteria		Revised El Escorial criteria	
RNA quality (RIN average +/− SEM)	Not specified		RIN ≥ 8		RIN ≥ 7	

**Table 8:** Random forest PC1 gene expression cluster performance metrics for neurodegenerative diseases (trained on AD and tested on PD or ALS).

Dataset	AUC	Sensitivity	Specificity	Precision
Parkinson’s disease (GSE6613)	0.48	0.69	0.30	0.69
Parkinson’s disease, drug naïve (GSE72267)	0.54	0.72	0.39	0.65
Amyotrophic lateral sclerosis (GSE112681)	0.50	0.55	0.47	0.73

**Table 9:** Random forest PC2 metabolic process cluster performance metrics for neurodegenerative diseases (trained on AD and tested on PD or ALS).

Dataset	AUC	Sensitivity	Specificity	Precision
Parkinson’s disease (GSE6613)	0.52	0.68	0.27	0.68
Parkinson’s disease, drug naïve (GSE72267)	0.61	0.71	0.45	0.83
Amyotrophic lateral sclerosis (GSE112681)	0.52	0.63	0.15	0.72

**Table 10:** Random forest PC1 gene expression cluster performance metrics for neurodegenerative diseases (trained and tested on same disease dataset).

Dataset	AUC	Sensitivity	Specificity	Precision
Parkinson’s disease (GSE6613)	0.44	0.64	0.5	0.78
Parkinson’s disease, drug naïve (GSE72267)	0.50	0.67	N/A	1.00
Amyotrophic lateral sclerosis (GSE112681)	0.89	0.91	0.71	0.82

**Table 11:** Random forest PC2 metabolic process cluster performance metrics for neurodegenerative diseases (trained and tested on same disease).

Dataset	AUC	Sensitivity	Specificity	Precision
Parkinson’s disease (GSE6613)	0.86	0.86	0.67	0.80
Parkinson’s disease, drug naïve (GSE72267)	1.00	1.00	1.00	1.00
Amyotrophic lateral sclerosis (GSE112681)	0.89	0.91	0.74	0.84

## Data Availability

All data in this study are publicly accessible in the Gene Expression Omnibus (GEO) database under accession numbers GSE97760, GSE63061, GSE63060, GSE6613, GSE72267, and GSE112681. All data generated from our analyses is available in the Supplementary Tables. Code is available on A.S.’s Github at https://github.com/Art83.

## Supplementary Materials

The supporting information can be downloaded at: https://www.mdpi.com/article/10.3390/ijms241915011/s1.
