# Supplementary material for: Blood-Based Transcriptomic Biomarkers Are Predictive of Neurodegeneration Rather Than Alzheimer’s Disease
Source: Int J Mol Sci. 2023 Oct 9;24(19):15011. doi: 10.3390/ijms241915011 (PMC10573468; doi:10.3390/ijms241915011)
Supplement: Supplementary file 1 [file ijms-24-15011-s001.zip › ijms-2641757-supplementary figures.pdf]

Blood-based transcriptomic biomarkers are predictive of neurodegeneration rather than Alzheimer's disease

Artur Shvetcov [1,2], Shannon Thomson [3,4], Jessica Spathos [3], Ann-Na Cho [5], Heather M. Wilkins [6,7,8], Shea J. Andrews [9], Fabien Delerue [10], Timothy A. Couttas [11], Jasmeen Kaur Isaar [12,13,14], Finula Isik [3,4], Simran Kaur [15,16], Eleanor Drummond [4,17], Carol Dobson-Stone [4,17], Shantel L. Duffy [18], Natasha M. Rogers [19.20.21], Daniel Catchpoole [22,23], Wendy A. Gold [4,12,13], Russell H. Swerdlow [6,7,8,24], David A. Brown [3,21,25], & Caitlin A. Finney [3,4*]

Supplementary Figures

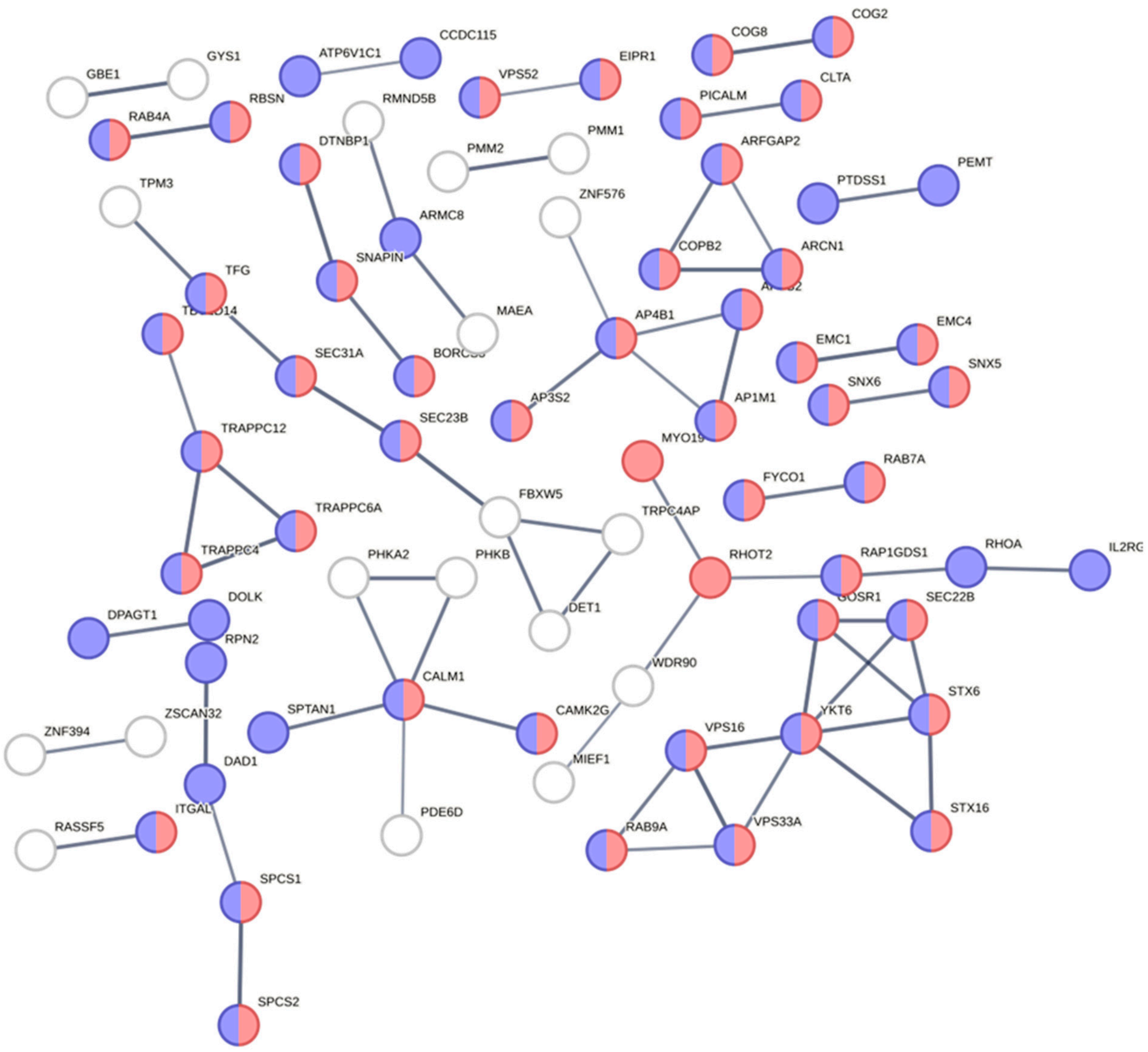

Supplementary Figure S1. Characterization of the biological function and cellular localization of component 1 (PC1) k-means cluster 1 (red) using Gene Ontology and STRING. The STRING network analysis includes the following interaction sources: experiments, databases, co-expression, neighbourhood, and gene fusion. The minimum interaction score was set to 0.7 (high confidence), and disconnected nodes in the network are hidden. The thickness of the line indicates the confidence in the interaction. The red nodes indicate genes involved in cellular localization (FDR = 3.61e-11), and the blue nodes indicate cellular localization of the endomembrane system (FDR = 6.02e-22).

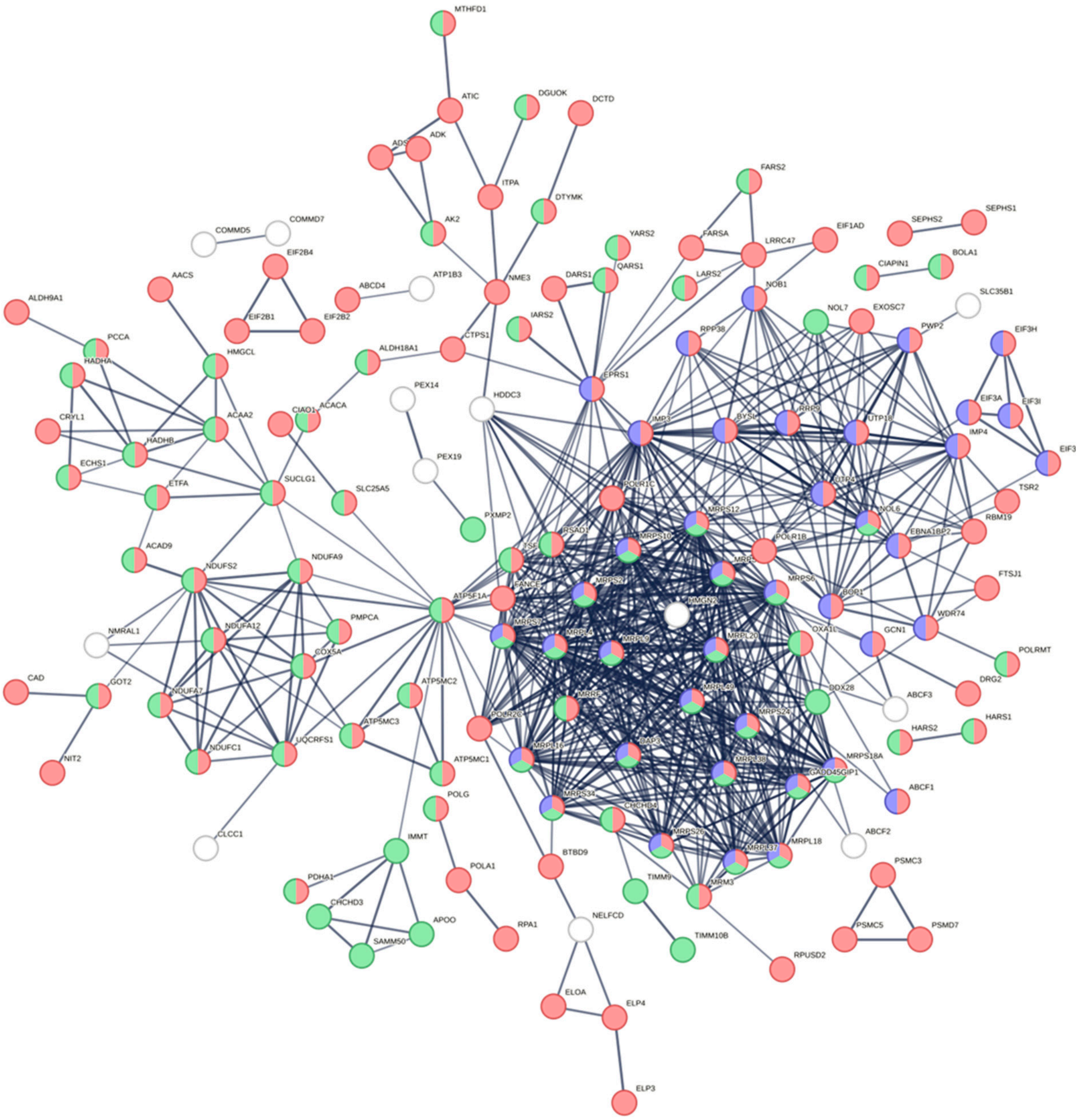

Supplementary Figure S2. Characterization of the biological function and cellular localization of component 1 (PC1) k-means cluster 2 (yellow) using Gene Ontology and STRING. The STRING network analysis includes the following interaction sources: experiments, databases, co-expression, neighbourhood, and gene fusion. The minimum interaction score was set to 0.7 (high confidence), and disconnected nodes in the network are hidden. The thickness of the line indicates the confidence in the interaction. The red nodes indicate genes involved in metabolic processes (FDR = 5.29e-25); the green nodes indicate cellular localization of the mitochondrion (FDR = 2.87e-40); the blue nodes indicate ribonucleoprotein complex (FDR = 5.93e-12).

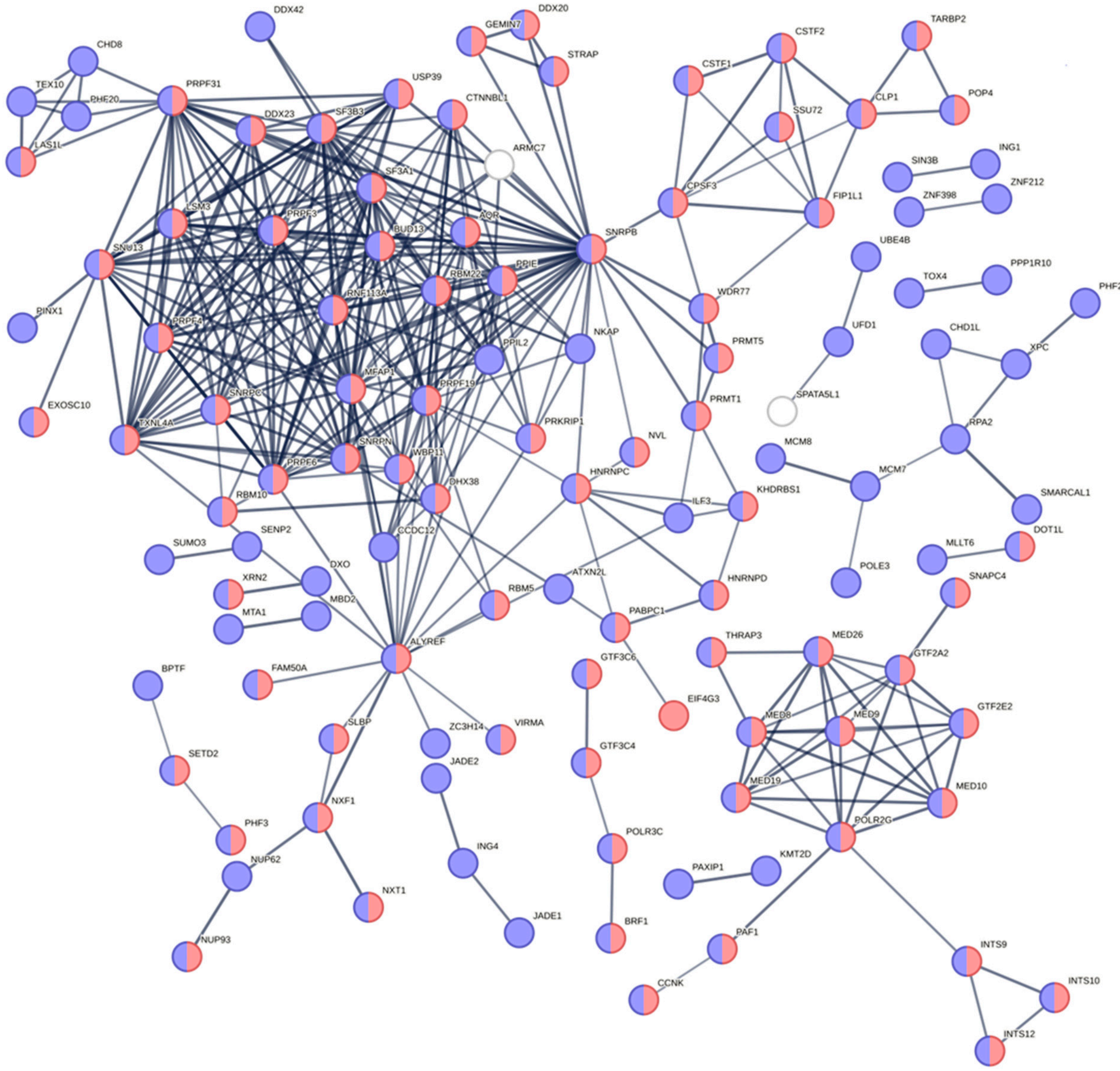

Supplementary Figure S3. Characterization of the biological function and cellular localization of component 1 (PC1) k-means cluster 3 (green) using Gene Ontology and STRING. The STRING network analysis includes the following interaction sources: experiments, databases, co-expression, neighbourhood, and gene fusion. The minimum interaction score was set to 0.7 (high confidence), and disconnected nodes in the network are hidden. The thickness of the line indicates the confidence in the interaction. The red nodes indicate genes involved in gene expression (FDR = 3.87e-28), and the blue nodes indicate cellular localization of the nucleus (FDR = 1.75e-44).

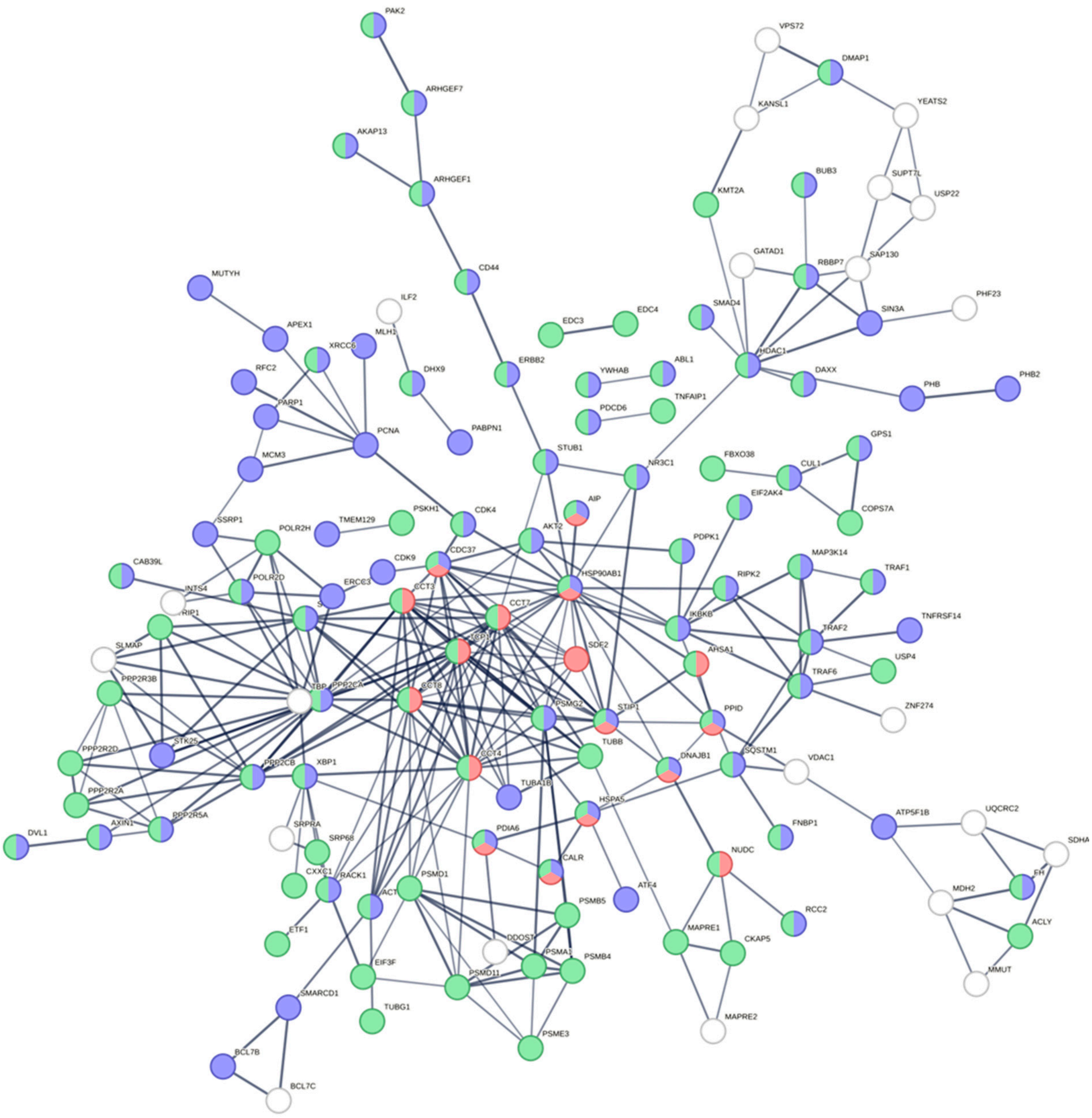

Supplementary Figure S4. Characterization of the biological function and cellular localization of component 1 (PC1) k-means cluster 4 (blue) using Gene Ontology and STRING. The STRING network analysis includes the following interaction sources: experiments, databases, co-expression, neighbourhood, and gene fusion. The minimum interaction score was set to 0.7 (high confidence), and disconnected nodes in the network are hidden. The thickness of the line indicates the confidence in the interaction. The red nodes indicate genes involved in protein folding (FDR = 2.80e-9); the blue nodes indicate genes involved in cellular response to stimulus (FDR = 1.69e-9); the green nodes indicate cellular localization of the cytosol (FDR = 1.19e-16).

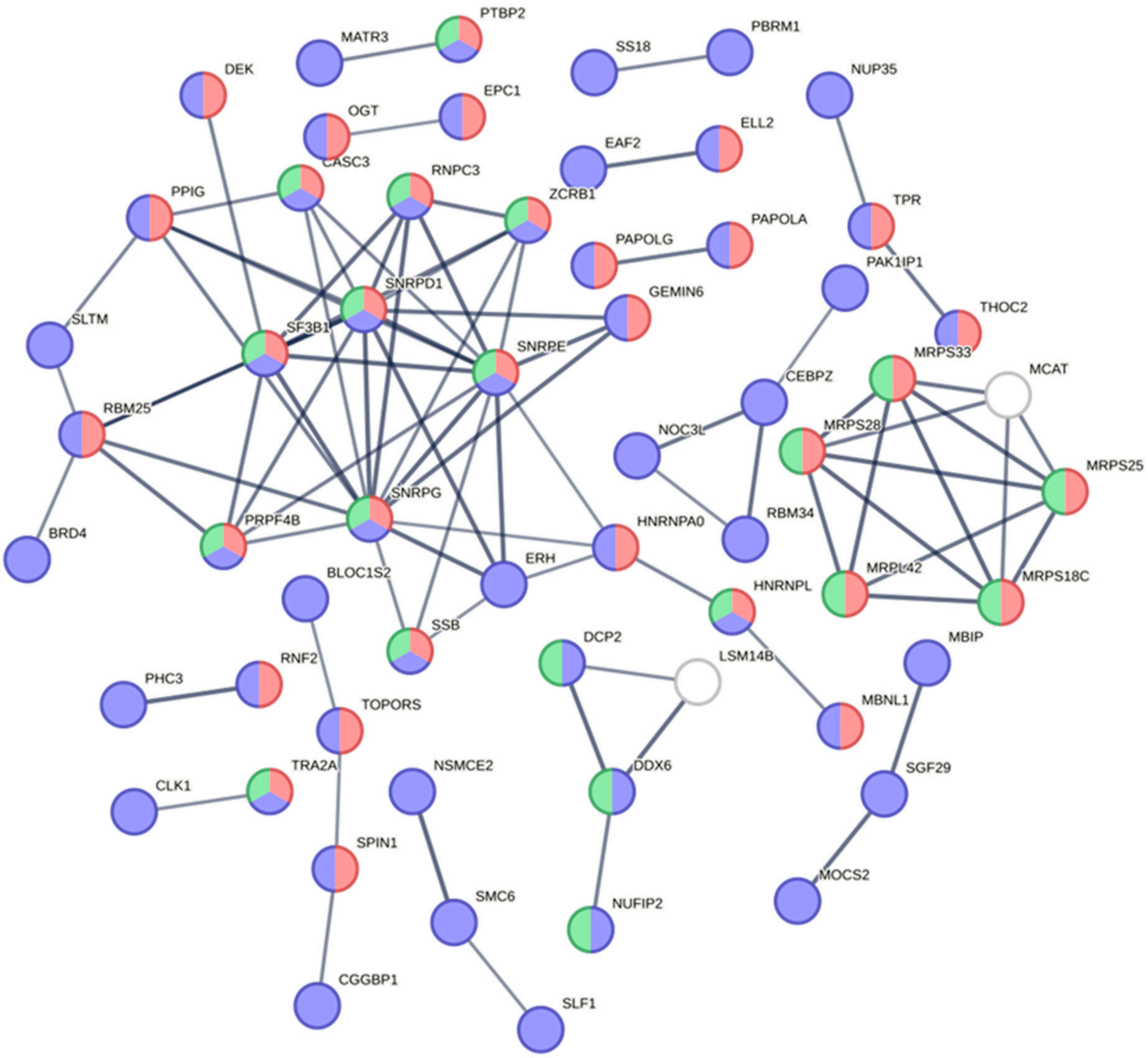

Supplementary Figure S5. Characterization of the biological function and cellular localization of component 1 (PC2) k-means cluster 1 (red) using Gene Ontology and STRING. The STRING network analysis includes the following interaction sources: experiments, databases, co-expression, neighbourhood, and gene fusion. The minimum interaction score was set to 0.7 (high confidence), and disconnected nodes in the network are hidden. The thickness of the line indicates the confidence in the interaction. The red nodes indicate genes involved in gene expression (FDR = 2.32e-9); the blue nodes indicate cellular localization to the nucleus (FDR = 1.83e-21); and the green nodes indicate cellular localization of the ribonucleoprotein complex (FDR = 0.00024).

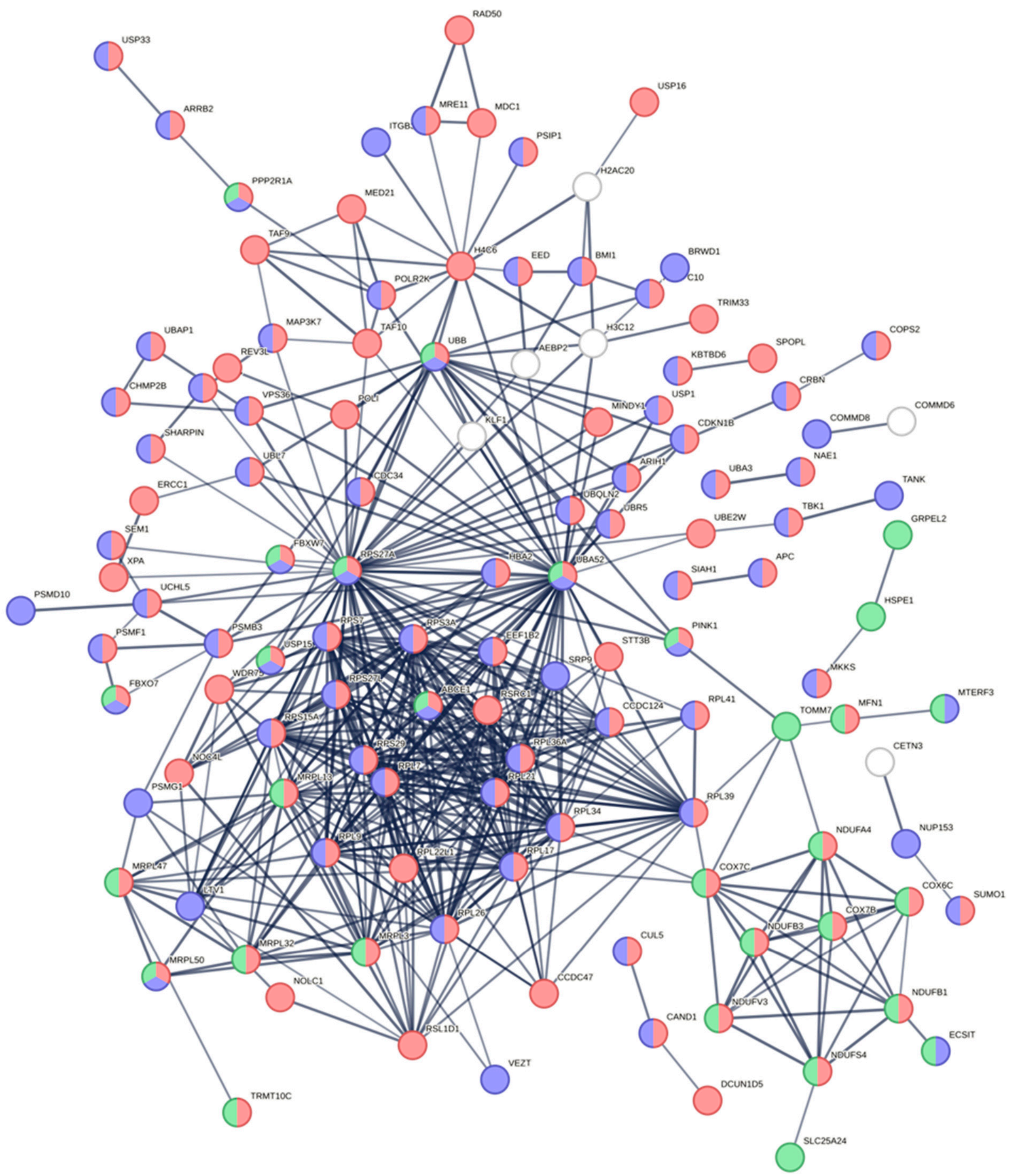

Supplementary Figure S6. Characterization of the biological function and cellular localization of component 1 (PC2) k-means cluster 2 (yellow) using Gene Ontology and STRING. The STRING network analysis includes the following interaction sources: experiments, databases, co-expression, neighbourhood, and gene fusion. The minimum interaction score was set to 0.7 (high confidence), and disconnected nodes in the network are hidden. The thickness of the line indicates the confidence in the interaction. The red nodes indicate genes involved in metabolic process (FDR = 2.58e-22); the blue nodes indicate cellular localization to the cytosol (FDR = 1.78e-16); the green nodes indicate cellular localization of the mitochondrion (FDR = 4.40e-6).

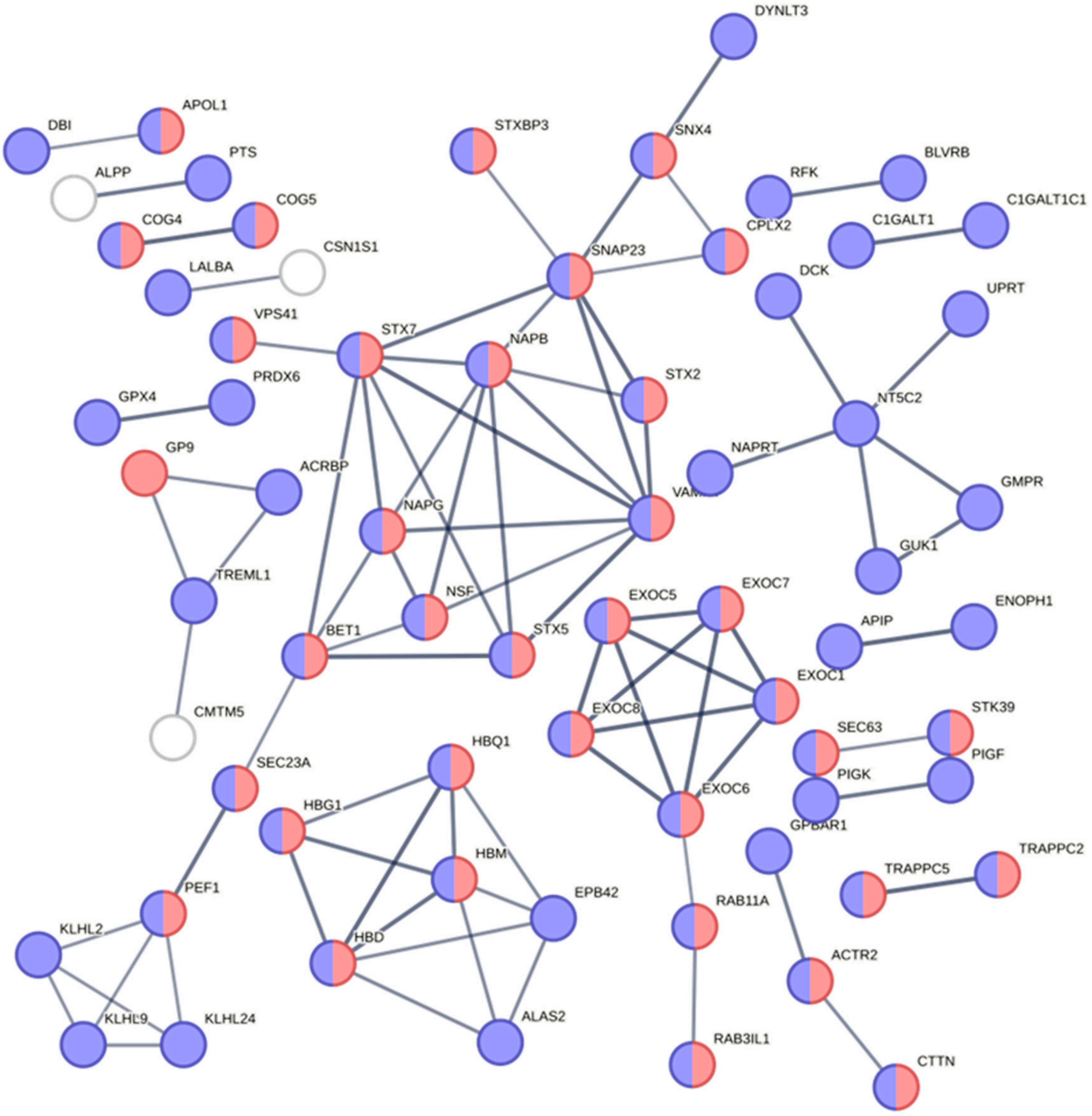

Supplementary Figure S7. Characterization of the biological function and cellular localization of component 1 (PC2) k-means cluster 3 (green) using Gene Ontology and STRING. The STRING network analysis includes the following interaction sources: experiments, databases, co-expression, neighbourhood, and gene fusion. The minimum interaction score was set to 0.7 (high confidence), and disconnected nodes in the network are hidden. The thickness of the line indicates the confidence in the interaction. The red nodes indicate genes involved in transport (FDR = 4.58e-6), and the blue nodes indicate cellular localization to the cytoplasm (FDR = 4.19e-10).

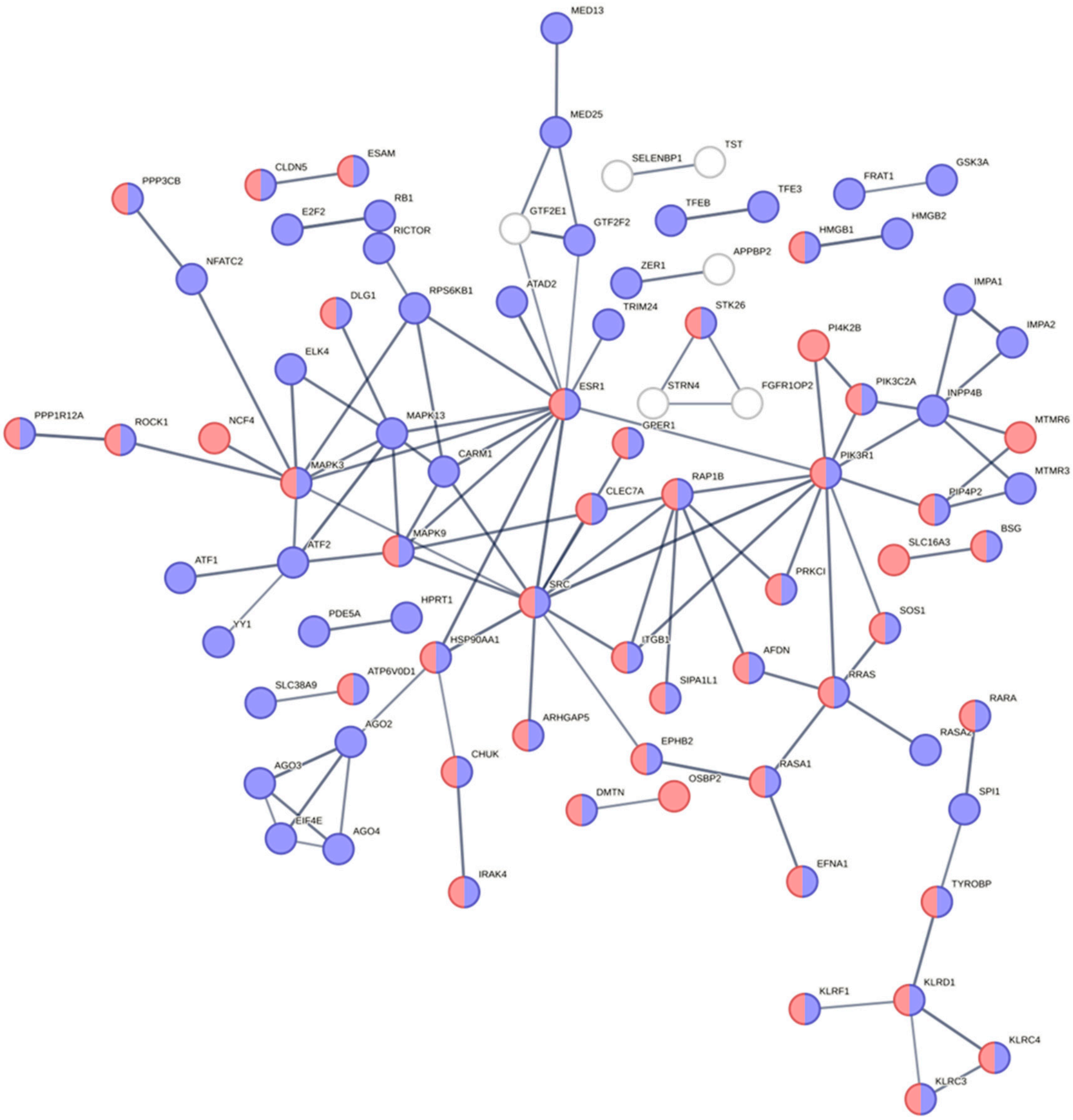

Supplementary Figure S8. Characterization of the biological function and cellular localization of component 1 (PC2) k-means cluster 4 (blue) using Gene Ontology and STRING. The STRING network analysis includes the following interaction sources: experiments, databases, co-expression, neighbourhood, and gene fusion. The minimum interaction score was set to 0.7 (high confidence), and disconnected nodes in the network are hidden. The thickness of the line indicates the confidence in the interaction. The red nodes indicate genes involved in the regulation of the cellular process (FDR = 2.49e-10), and the blue nodes indicate cellular localization to the plasma membrane (FDR = 0.00062).
